# Oil-in Water Vegetable Emulsions with Oat Bran as Meat Raw Material Replacers: Compositional, Technological and Structural Approach

**DOI:** 10.3390/foods12010040

**Published:** 2022-12-22

**Authors:** Ana M. Herrero, Esther Merino, Irene Muñoz-González, Claudia Ruiz-Capillas

**Affiliations:** Institute of Food Science, Technology and Nutrition (CSIC), José Antonio Novais 10, 28040 Madrid, Spain

**Keywords:** oat bran, olive oil, oil–in–water emulsion, bioactive compounds, meat replacer, technological properties, structural characteristics

## Abstract

The unique composition and technological properties of some oat bran components (mainly protein and soluble fiber) and olive oil make them a good choice to form oil-in-water vegetable emulsions. The different concentrations of oat bran were studied to form olive oil-in water (O/W) emulsions to apply as a replacement for fat and meat. As a result, four O/W emulsions (OBE) were formulated with 10% (OBE10), 15% (OBE15), 20% (OEB20), and 30% (OBE30) oat bran concentrations and 40% olive oil, with the corresponding amount of water added for each O/W emulsion. Composition, technological properties (thermal stability, pH, texture), and lipid structural characteristics were evaluated. The results showed that low oat bran content (OEB10)—with a lower concentration of oat protein and β-glucans—resulted in an O/W emulsion with an aggregated droplet structure and lower thermal stability and hardness. These connections between composition, technology, and structural properties of olive O/W emulsions elaborated with oat bran could help in making the optimal choice for their potential application in the production of foods such as healthier meat products.

## 1. Introduction

Oil-in-water (O/W) emulsions involve at least two immiscible phases where oil is the dispersed phase, as tiny droplets, in the aqueous continuous phase. These types of emulsions have three parts: the oil inside the droplets; the interfacial material between lipids and the aqueous phase; and the aqueous phase itself. However, O/W emulsion-based foods are thermodynamically unstable systems that tend to separate into immiscible phases over time. The interfacial material can consist of emulsifiers and/or stabilizers such as proteins or small molecules that enhance emulsion stability [1,2,3]. The O/W emulsions in food can be end products themselves (mayonnaise, cream, etc.) or can be employed as ingredients participating in the formation of more complex food structures. The O/W emulsions that are used as animal fat replacers to obtain healthier lipid profiles in meat products are an example of the latter, i.e., ingredients [4,5,6].

Several different protein sources and vegetable and marine oils and/or combinations of these have been employed to form O/W emulsions with various compositions and technological properties that can be used as animal fat replacers in meat products [4,6,7,8,9]. Olive is the vegetable oil employed for this function that has attracted the most attention as it is a source of monounsaturated fatty acids and antioxidants and has a positive impact on human health [10].

The use of proteins as emulsifiers and/or stabilizers in O/W emulsions formed from oats could be an attractive option (*Avena sativa* L.). It is a good source of plant protein (between 12 and 25% in groats) and is high-quality in terms of the amino acid composition of that protein [11,12]. Therefore, its high protein content and functionality make it an attractive alternative to animal protein. In addition, oats also feature other components such as unsaturated fatty acids, antioxidants, and soluble fiber, which, together with protein, give this food interesting nutritional and technological properties for the formation of O/W emulsions. It is worth mentioning the role played by protein and soluble fiber in oats. The major storage proteins in oats are salt-soluble globulins, which account for 70–80% of the total proteins [13]. Oat proteins in general feature good solubility and emulsifying properties [11]. Oats have more soluble fiber than any other cereal, resulting in slower digestion and an extended sensation of fullness. In addition, whole-grain soluble fibers of oats contain a type of polysaccharide known as β-glucans, which have numerous health benefits [14,15]. Furthermore, β-glucans also play an important technological role in the production of processed foods [16,17]. Some researchers have discovered that fiber hydroxyl groups, including β-glucans, interact with water via hydrogen bonding, increasing their ability to absorb and/or retain water [16,18]. This could modify their water absorption properties and bring about alterations in gelatinization and the thermal and rheological characteristics of the foods to which they are added [19,20]. Oat bran, a by-product of oat flour milling, has specifically been employed as a fiber-enriching ingredient but is also high in protein. This unique composition of oat bran makes it challenging to take full advantage of all its properties. Considering oat bran’s unique composition and the technological properties of its components, one of the potential uses is in the formulation of O/W emulsions with optimal nutritional and technological properties, including stability (without phase separation, appropriate water and fat binding properties, optimal textural behavior, etc.) [1,2,21,22]. It has been demonstrated by numerous studies that oat bran extracts from food by-products can be employed as a natural emulsifier [22,23]. Moreover, O/W emulsions using modified oat bran containing deamidated oat protein are a good ingredient for creating stable emulsions [24]. Additional work has been done with O/W emulsions using oat and different cold gelling agents to form emulsion gels with optimal nutritional and technological properties applied as animal fat replacers [25]. However, to the best of our knowledge, no study has yet been conducted on O/W emulsions used as fat and meat raw material replacers, stabilized only with unmodified oat bran, which has the added advantage of being very easy to work with. It would also be helpful to gain a deeper understanding of the structure and interactions between the different components affecting these O/W emulsions, with kinetic stability being a particularly important aspect [26].

Hence, the main aim of this study was to create olive O/W emulsions using different concentrations of oat bran to determine which confers the appropriate composition and greatest technological stability. The research intendeds to shed light on the causal relationship between composition and technological and structural properties to better understand their stability process. To this end, we employed Attenuated Total Reflectance (ATR) Fourier Transform Infrared spectroscopy (FTIR), as this is a fast and non-destructive analytical tool capable of simultaneously offering detailed structural information on emulsion components. The end goal is to develop oat-bran emulsions for future application in meat products as meat replacers.

## 2. Materials and Methods 

### 2.1. Materials

Oat bran powder (OatWell 22%), Zeus Química, S.A. Barcelona, Spain; composition (according to the supplier): 20% protein, 20% carbohydrates, 44% total dietary fiber (22% of which is β-glucan soluble fiber), and 5.1% fat; physical properties (according to the supplier): very light yellow-brown colour, neutral taste, fine texture “fluffy” fiber particles, bulk density 400 g per litre (±50 g/litre), particle size 35–300 microns, granulation < 250 μm/(50% < 125 μm). Olive oil (Carbonell Virgen Extra, SOS Cuétara S.A., Madrid, Spain).

### 2.2. Preparation of Olive O/W Emulsions with Oat Bran

**Figure 1:**
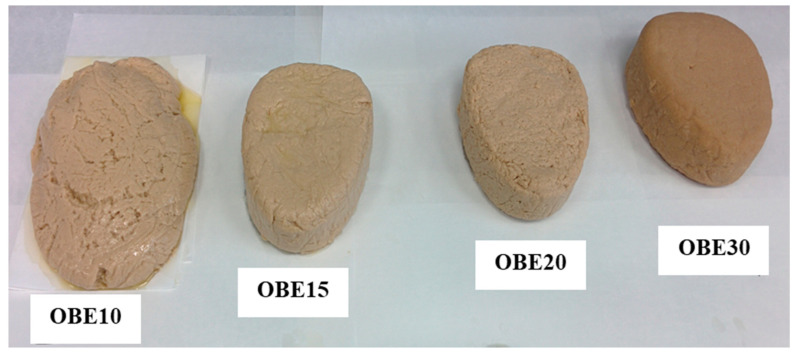
Typical appearance of different oat bran O/W emulsions formulated with 10% (OBE10), 15% (OBE15), 20% (OEB20), and 30% (OBE30) oat bran. See Table 1 for a sample description.

The four vegetable O/W emulsions were prepared with different concentrations of oat protein, i.e., different amounts of oat bran (Table 1). The olive oil content was the same for all (40%). Specifically, the four oat O/W emulsions (OBE) were prepared with the following concentrations of oat bran: 10% (OBE10), 15% (OBE15), 20% (OEB20), and 30% (OBE30) (Table 1). These O/W emulsions were made by homogenizing (30 s at 5600 rpm, approx.) (Thermomix TM 31, Vorwerk España M.S.L., S.C., Madrid, Spain) the corresponding quantities of water and oat bran (Table 1). The appropriate quantity of olive oil was then slowly added to each sample (Table 1) while the homogenizer was running (approx. 5600 rpm) [27]. Each homogenized mixture of water, olive oil, and oat bran was then poured directly into a metal recipient under pressure and stored at 2 °C for 24 h to achieve the final emulsion. These vegetable O/W emulsions were then analyzed. Typical vegetable emulsions can be viewed in Figure 1.

### 2.3. Protein Content

The protein content was measured in quadruplicate with an LECO FP-2000 Nitrogen Analyzer (Leco Corporation, St. Joseph, MI, USA).

### 2.4. Technological Properties

#### 2.4.1. Total Fluid Release (TFR) and pH Analysis

The thermal stability, i.e., water and fat binding properties, of the emulsions was determined by identifying the total fluid release (TFR) during heating [28]. A measurement of 30 g of each emulsion were packed into hermetically sealed tubes, which were then heated in a water bath for 30 min at 70 °C. The tubes were then opened and left standing upside down for 50 min to release fluid (composed of fat and water) onto a plate. The TFR was the percentage of the initial sample weight. Measurements were carried out in triplicate for each type of vegetable emulsion. 

The pH of each O/W emulsion type was analyzed in triplicate using an 827 Metrohm pH-meter (Metrohm AG, Herisau, Switzerland) at room temperature. The emulsions were placed in distilled water at a ratio of 1:10 *w/v*.

#### 2.4.2. Penetration Test

The penetration tests were carried out on a TA-XT plus Texture Analyzer (Texture Technologies Corp., Scarsdale, NY, USA) [29]. A total of six measurements were made for each emulsion type. The penetration force (PF, N) was the maximum force along the force–deformation curve.

### 2.5. Structural Characteristics

#### Attenuated Total Reflectance (ATR)-FTIR Spectroscopy Analysis

The infrared spectra were determined (mid-infrared mode) for each type of O/W emulsion using a Perkin-Elmer SpectrumTM 400 spectrometer (Perkin Elmer Inc.,Tres Cantos, Spain) equipped with an ATR sampling device [28,29]. The measurements were taken at room temperature using approximately 1 g of the samples (without any prior sample treatment), placed directly on the surface of the ATR crystal. A total of Nine determinations were made per emulsion type. A total of three sum spectra (72 accumulations) were performed for each O/W emulsion type.

### 2.6. Statistical Analysis

The analysis of variance (ANOVA) was performed to assess the statistical significance (*p* < 0.05) of the effect of emulsion formulation using IBM SPSS Statistics 22 (SPSS Inc., Chicago, IL, USA). In addition, the least squares differences method was applied to compare the mean values of formulations, while Tukey’s HSD test was used to detect the significant differences (*p* < 0.05) between the formulations. The Pearson’s product-moment correlation coefficient, r, was performed using Statgraphics Plus version 5.0 software (Statgraphics Technologies, Inc. Virginia United States) to establish the correlation between oat bran content in emulsions and technological and structural properties.

## 3. Results

### 3.1. Composition and Energy Value

To consider the viability of using these oat O/W emulsions as meat raw material replacers, we first needed to evaluate the most important parts of their composition. For example, from OBE10 to OBE30, the protein content ranged from 2.02 to 5.95% due to the different quantities of oat bran incorporated into the vegetable emulsion (Table 1). Oats have a higher level of quality protein and a better amino acid profile than most other cereals due to their high levels of lysine and threonine [30]. Oat protein contains globulin (the major storage portion, 50–80%), prolamins (avenins, 4–15%), albumin (1–12%), and glutelin (10%) [11]. Oats have a protein that is gluten-free, which means that people suffering from celiac disease can safely consume oat protein-based products [11] such as the emulsions indicated (OEA10, 15, 20, and 30).

The total dietary fiber (TDF), theoretically classified as fiber, is an important part of a complete diet, with the daily recommended intake for adults being 28–36 g/day [31]. The TDF content in these samples ranged from 4.4 (OEB10) to 13.2% (OEB30), correlating with oat bran content (Table 1). According to EU Regulation 1924/2006, these O/W vegetable emulsions and the products they are added to as meat replacers can make the nutritional claim that said products are a “source of fiber.” According to that regulation, this claim can be made if the product includes a minimum of 3 g of fiber per 100 g of product or at least 1.5 g of fiber per 100 kcal, and can claim “high fiber content” if the product contains at least 6 g of fiber per 100 g or 3 g of fiber per 100 kcal. On the basis of the theoretical TDF values, the expected β-glucan soluble fiber content of the vegetable emulsions would be approximately: OEB10 = 0.97%, OEB15 = 1.45%, OEB20 = 1.94%, and OEB30 = 2.90%. The ability of β-glucan to help maintain normal blood cholesterol levels has led to health claims [32]. This latter health claim only applies to foods that contain at least 1 g of β-glucans per quantified portion from oat, oat bran, barley and barley bran or mixtures of these β-glucans. However, this claim may be true only if the product label informs consumers that the beneficial effect is obtained by consuming 3 g of β-glucans per day from oat, oat bran, barley, barley bran, or a combination of these [32]. A number of researchers have claimed that β-glucans can significantly reduce the incidence of certain diseases, such as cardiovascular diseases and the symptoms of diabetes [33,34].

The principal source of fat in the vegetable emulsions formulated is olive oil (Table 1) and contains 14.87% saturated fatty acids (SFA), 75.32% monounsaturated fatty acids (MUFA), and 8.97% polyunsaturated fatty acids (PUFA) [35].

In addition to these characteristics, the energy value of these vegetable emulsions, compared to the energy value of the animal fat typically used in the manufacture of meat products, must be considered if they are to be used as animal fat replacers: 9 kcal/g for fat; 4 kcal/g for protein and carbohydrates; and 2 kcal/g for dietary fiber. The expected energy values of the emulsions were 389.5, 403.72, 419.3, and 447.97 kcal/g for OEB 10, 15, 20, and 30, respectively. This means that, compared to the approximate energy value of 673 kcal/100 g for pork backfat typically employed in meat products, calorie reduction would range from 42% (OEB30) to 33% (OBE10) depending on which of the O/W emulsions is used as the fat replacer.

### 3.2. Technological Properties

#### 3.2.1. Total Fluid Release (TFR) and pH Analysis

While there was no considerable phase separation or disturbance of physical structure after processing and heating in any of the vegetable O/W emulsions, significant differences were obtained in TFR values (Table 2). There was no substantial TFR (*p* > 0.05) detected in the emulsion with the highest oat bran content (OBE30), with the highest TFR values (*p* < 0.05) corresponding to the emulsion with the lowest oat bran amount (OBE10) (Table 2). These data show a significant negative correlation between thermal stability (water and fat binding), TFR (r = −0.7837, *p* < 0.026), and the emulsion’s oat bran content. This suggests that oat bran has a very relevant function in the creation and stabilization of these O/W emulsions by retaining water and oil. Oat protein and β-glucans have previously been shown to have functional properties such as gelling and emulsifying activity, water retention, and fat binding capacity [11].

The pH data for each of the oat bran vegetable emulsions are shown in Table 2 and indicate that the more oat bran (*p* < 0.05) in the emulsions, the lower the pH. A number of researchers have detected that dairy products or emulsions fortified with β-glucans have a significantly lower pH [25,36], which would explain the negative correlation between pH and oat bran concentration in the emulsions. These vegetable emulsion pH data (Table 2) are within the ranges described for pork backfat, another aspect indicating their appropriateness as animal material replacers in foods such as meat derivates [37].

#### 3.2.2. Penetration Test

Regarding textural behavior, all vegetable O/W emulsions exhibited a strong breaking point, enabling the precise measurement of their PF. The Table 2 indicates that there was a significant positive correlation between PF value and oat amount (*p* < 0.05). The oat bran emulsions have different oat/fat ratios, which could influence their characteristics, resulting in an increase in the content of oat bran leading to a rise in the “effective” concentration of oat protein in the emulsion. The increase in protein concentration in the continuous phase of the vegetable O/W emulsion generally gave rise to an increase in the number of locations on the polypeptide chains capable of interacting, which could account for products having firmer structures [25].

The different oats/oil ratios reflect the presence of β-glucans in oat. According to research on the structural/functional property relationship of cereal β-glucans, these hydrocolloids can create gel networks in aqueous media and the rate and level of gelation correlate to the molecular size and fine structure of the polysaccharide [38,39]. An increase in strength and a decrease in brittleness of oat gel with increasing concentrations and molecular weight of polymer β-glucans were also found [40]. The β-glucan content has also been linked to an increase in gelation rate [39]. when cereal β-glucan concentrates are added to some foods, such as low-fat cake or low-fat white-brined cheese, become even harder when concentrates are incorporated [36,41]. This could be due to the emulsion stabilizing and gelling properties of oat β-glucan. A number of researchers have demonstrated that barley β-glucan contributes to the formation of a gelled network. [42] also related to a firmer texture.

### 3.3. Structural Characteristics

#### Attenuated Total Reflectance (ATR)-FTIR Spectroscopy Analysis

**Figure 2:**
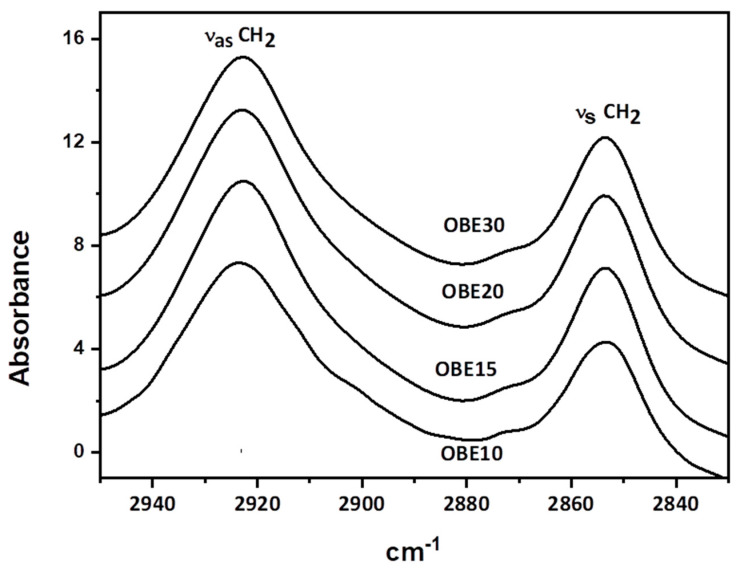
ATR-FTIR spectra in the 2950–2830 cm^−1^ region of oat bran O/W emulsion. See Table 1 for sample description.

The 2950–2830 cm^−1^ spectral region (Figure 2) was examined to establish the lipid structure of the different oat bran emulsions (OB10, 15, 20, and 30). When there is the isolation of the spectral effect of lipids in this spectral region, the spectral influences of water and other ingredients employed to formulate the emulsion were eliminated [28,29]. The 2950–2830 cm^−1^ spectral region was characterized by two clear bands at approximately 2923 and 2854 cm^−1^ (Figure 2) causing stretching vibrations of the acyl CH_2_ groups of lipids from the asymmetric (ν_as_ CH_2_) and symmetric (ν_s_ CH_2_) respectively [43,44,45].

The modifications in the broadening of these infrared bands usually account for changes in the conformational order of the lipid acyl chains and their dynamics [46,47]. These modifications could be associated with lipid interactions with other biomolecules, such as proteins, which normally trigger spectral alterations of the methylene ν CH modes of lipid chains, more noticeable in the asymmetric bands (ν_as_ CH_2_) than in the symmetric (ν_s_ CH_2_) bands. The broadening of these infrared bands is typically detected by increases in their half-bandwidth [47]. The Table 3 shows the half-bandwidths of the ν_as_ CH_2_ and ν_s_ CH_2_ bands of oat bran O/W emulsions (OA10, 15, 20 and 50) and pure liquid olive oil as a control. The data show broadening (*p* < 0.05), i.e., higher half-bandwidth values of the ν_as_CH_2_ band (at approx. 2923 cm^−1^) of all vegetable emulsions when compared with pure olive oil (Table 3). This could be due to a decrease in the conformational order of the lipid acyl chains and their increased dynamics [47] resulting from the fact that in the creation of emulsions, oil acyl chains interact with structurally different acyl and protein chains and other interactions such as intermolecular connections between oil acyl chains and water and/or other compounds [47]. Hence, the creation of the emulsion apparently produces disorder in the olive oil lipid chain due to lipid interactions (protein–lipid, lipid–water, etc.).

Numerous studies have shown that oat proteins occur as colloidal aggregates, and some oat proteins, such as oat globulins, may create aggregates at the interface [48]. The stability of protein aggregates (particles) in liquid media seems to be a decisive factor in their emulsifying and/or gelling capacity [48]. In addition, a comparison of the half-bandwidths of the ν_as_ CH_2_ (2923 cm^−1^) of all oat emulsions (OB10, 15, 20, and 30) (Table 3) show that the lower the oat concentration, the higher the half-bandwidths, and, hence, the greater the lipid chain disorder or lipid interactions. Based on data described in the literature [28,49], this coincides with the increased occurrence of the characteristic structural state of oat proteins based on aggregates in vegetable O/W emulsions with lower oat bran content (OB10). This structural behavior, based on a greater number of aggregates, corresponds to less hardness and stability, since it is well known that physical aggregation in an emulsion also influences its stability [26,28]. Based on these findings, the lower the oat content, the more the emulsion are based on aggregated droplets associated with less hardness and stability in terms of higher water and fat loss (determined as TFR). It is therefore safe to say that there is a significant negative correlation between oat content, lipid interaction (in terms of the broadening of ν_as_ CH_2_) (R = −0.823, *p* < 0.0009) and TFR (R = −0.784, *p* < 0.0000), and a significant positive correlation with hardness (R = 0.987, *p* < 0.0026) in the oat bran O/W samples. These correlations between the technological and structural properties of oat bran emulsions could help in choosing the most optimal emulsion for potential in the production of food, such as healthier meat products. For example, some oat emulsion gels have already been used as new animal fat replacers in the development of healthier fresh sausages [50]. While cooking processes such as grilling did modify some of their technological properties, for the most part, all animal fat and oat emulsion sausages behaved similarly [51].

## 4. Conclusions

This study highlights possible advances from developing vegetable O/W emulsions that can be employed as meat raw material (fat and meat) replacers made only from plant-derived ingredients with an aqueous phase containing protein from oat bran and a lipid phase comprising vegetable oil, in this case olive oil. Due to the special composition of oat bran and olive oil, these vegetable O/W emulsions contain a variety of bioactive compounds [high-value proteins, soluble fiber (β-glucans), MUFA, etc.]. The concentration of these bioactive compounds in the O/W emulsions depends on the quantity of these ingredients used in their formulation. Additionally, these vegetable emulsions feature the technological properties needed for their use as meat raw material replacers. Several the technological properties of these vegetable O/W emulsions, such as texture, water content, and fat binding, depend mostly on the concentration of oat bran employed in their formulation.

The precise information concerning the structure of olive O/W emulsions stabilized with oat bran has been clarified by ATR-FTIR. This spectroscopic technique has furnished helpful information about olive oil lipid chain order/disorder linked to minor/major protein-lipid interactions related to the major/minor occurrence of aggregates. Specifically, O/W emulsions with a lower concentration of oat bran have a structure characteristic of oat proteins based on aggregates. These structural characteristics appear to define technological properties such as texture, water content, and fat binding capacity.

In these emulsions, an understanding of the relationships between the optimal composition, which confers certain technological and structural properties, will aid in the selection of the most suitable conditions for their viability as healthy plant-based ingredients in the production of food such as healthier meat products. Lastly, depending on the quantity of these healthy additives, the products comprising them may qualify for nutritional and health claims under EU regulations.

## Figures and Tables

**Table 1:** Formulation (%) and protein content (%) of different oat bran O/W emulsions.

Samples	Olive Oil (%)	Oat Bran (%)	Water (%)	Protein Content * (%)
OBE10	40	10	50	2.02 ± 0.27 ^d^
OBE15	40	15	45	2.91 ± 0.43 ^c^
OBE20	40	20	40	4.13 ± 0.51 ^b^
OBE30	40	30	30	5.95 ± 0.38 ^a^

* Different superscript letters in the same column indicate significant (*p* < 0.05) differences.

**Table 2:** Total fluid release (TFR, %), pH values, and penetration force (PF, N) of oat bran O/W.

Samples *	TFR (%)	pH	PF (N)
OBE10	12.13 ± 0.28 ^a^	7.11 ± 0.04 ^a^	0.11 ± 0.01 ^d^
OBE15	2.45 ± 0.66 ^b^	7.14 ± 0.07 ^a^	0.42 ± 0.04 ^c^
OBE20	0.27 ± 0.03 ^c^	7.05 ± 0.02 ^b^	1.09 ± 0.08 ^b^
OBE30	0.00± 0.00 ^d^	7.06 ± 0.02 ^b^	1.63 ± 0.07 ^a^

Mean ± standard deviation. Different superscript letters in the same column indicate significant (*p* < 0.05) differences. * For sample description see Table 1.

**Table 3:** Half-bandwidths of the ν_as_ CH_2_ (approx. 2923 cm^−1^) and (ν_s_ CH_2_) (approx. 2853 cm^−1^) bands of pure liquid olive oil and oat bran emulsions.

Samples *	Half-Bandwidth ν_as_ CH_2_	Half-Bandwidth ν_s_ CH_2_
Olive oil	27.2 ± 0.2 ^d^	15.8 ± 0.1 ^a^
OB10	29.9 ± 0.3 ^a^	16.6 ± 0.9 ^a^
OB15	28.7 ± 0.1 ^b^	16.4 ± 0.1 ^a^
OB20	28.2 ± 0.3 ^c^	16.2 ± 0.1^a^
OB30	28.0 ± 0.3 ^c^	16.3 ± 0.1 ^a^

Different superscript letters in the same column indicate significant (*p* < 0.05) differences. * For sample description see Table 1. Means ± standard deviation.

## Data Availability

The data is included in the article.
